# Local Thrombolysis for Successful Treatment of Acute Stroke in an Adolescent with Cardiac Myxoma

**DOI:** 10.1100/tsw.2011.90

**Published:** 2011-04-19

**Authors:** Natig Gassanov, Amir M. Nia, Kristina M. Dahlem, Stefan Ederer, Inga Wedemeyer, Evren Caglayan, Erland Erdmann, Fikret Er

**Affiliations:** ^1^ Department of Internal Medicine III, University of Cologne, Germany; ^2^ Institute of Pathology, University of Cologne, Germany

**Keywords:** atrial myxoma, myxoma-induced stroke, cardiac embolic source, thrombolysis

## Abstract

Intracardiac myxomas are the most common benign cardiac tumors in adults. They are a rare source of cardiogenic embolisms and sudden death, especially in young patients. This report describes the case of a male adolescent who presented with right-sided paresis and aphasia. Magnetic resonance imaging of the brain revealed an ischemic stroke without evidence of acute bleeding. Intra-arterial local thrombolysis was immediately started. An echocardiographic screening after successful thrombolysis with a remarkable recovery of symptoms detected a thrombotic-like mass in the left atrium. The mass was excised surgically, confirmed as a benign atrial myxoma, and the patient was discharged with restitution ad integrum. Thus, contrary to some critical reports, thrombolytic therapy for acute ischemic strokes due to atrial myxomas may be safe and highly effective.

A 17-year-old male was admitted with acute manifestation of right-sided hemiplegia and a sensory aphasia for approximately 1 h. His blood pressure was elevated to 150/95 mmHg, breathing and heartbeat rate were normal. Further, there was no history of trauma at the time of onset, no history of seizures, and no history suggestive of raised intracranial tension. The emergency magnetic resonance imaging (MRI) revealed an acute infarction due to thrombosis in the left medial cerebral artery territory. Consequently, local intra-arterial thrombolysis with recombinant tissue plasminogen activator was performed. The neurological deficits resolved completely.

For causal screening purposes, transthoracic echocardiography (TTE) was initiated and uncovered a striking mass (3.5 × 3.9 cm) in the left atrium (LA), which originated from the anterior mitral leaflet, with diastolic prolapse into the left ventricular (LV) cavity (Fig. 1A–D). The mitral valve was not obstructed hemodynamically. In accordance, the patient underwent successful surgical excision of the tumor. Histology confirmed the diagnosis of an atrial myxoma (Fig. 2A–D). The patient recovered appropriately without any complications and was discharged to neurological rehabilitation on the 10$^{th}$ day after surgery.

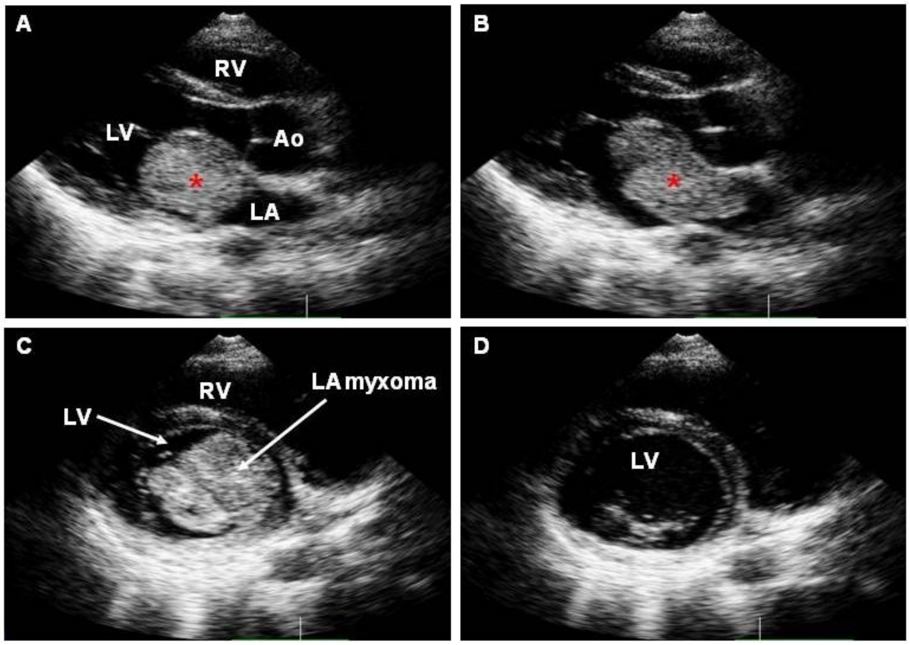

**FIGURE 1.** (A,B) Echocardiographic images of left atrial myxoma in parasternal long axis view (A, early diastolic; B, late diastolic). (C,D) Diastolic prolapse of the myxoma through the mitral valve (short axis view). LA, left atrium; LV, left ventricle; RV, right ventricle; Ao, Aorta, asterisk indicates myxoma.

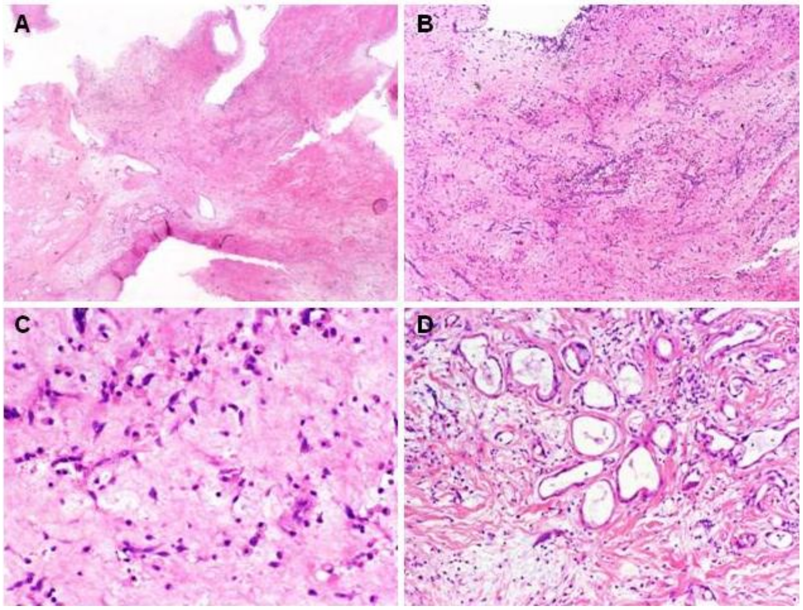

**FIGURE 2.** (A–D) Histology of the atrial myxoma. The tumor shows a lobulated and frond-like surface, and an abundant myxoid stroma with single spindle and stellate cells. Also present are some inflammatory cells (A, HE 20×; B, HE 40×). The spindle and stellate myxoma cells contain eosinophilic cytoplasm and uniform round or oval nuclei (C, HE 200×). In some areas, the myxoma shows foci of glandular differentiation (D, HE 100×).

Atrial myxomas are the most common primary cardiac tumors that are particularly frequent between the third and sixth decades of life[1]. They arise from multipotential mesenchymal cells of endocardial origin and commonly attach at the border of the fossa ovalis in the LA[2,3]. Systemic embolization is a frequent complication of cardiac myxomas, which often occurs in the cerebrovascular and retinal arteries[4]. A high rate of systemic embolization has been reported in 30–45% of patients with LA myxomas and up to 64% due to myxomas localized in the LV cavity[5]. Overall, cardiac myxomas are estimated to cause up to 0.5% of ischemic strokes, particularly if they are located in the LA[6].

Atrial myxomas often remain asymptomatic prior to an embolic event. Once detected, the surgical treatment should be considered because of the elevated risk of embolization. The surgical resection is the treatment of choice due to the low procedural mortality and good outcome[5].

Only a few reports have mentioned the use of thrombolytics in acute strokes caused by cardiac myxoma[7,8,9]. Currently, there are no guidelines or reliable studies on the role of thrombolytic agents and their application route in myxoma-induced strokes. Intravenous thrombolysis has been reported to be safe and effective in some cases[7,9]. However, it has been suggested to consider known myxoma as a relative contraindication to intravenous thrombolytic therapy for ischemic stroke because of a greater risk of hemorrhage from potential occult tumor emboli or microaneurysms[7]. Thus, subarachnoid hemorrhage from rupture of an aneurysm secondary to myxoma has been reported to aggravate clinical decision process[10].

Our single observation suggests that intra-arterial thrombolysis might be an effective and adequate option for acute treatment for cardiac myxoma-induced strokes. However, this is a single report, and much more information and many more cases are required before any change to treatment guidelines should be considered.
